# Sunbeds and skin cancer risk: quantifying a baseline estimate of sunbed facilities in South Africa prior to implementation of sunbed regulations

**DOI:** 10.11604/pamj.2017.26.188.10176

**Published:** 2017-03-30

**Authors:** Caradee Yael Wright, Patricia Nicole Albers, Anthony Ivor Reeder, Angela Mathee

**Affiliations:** 1 Environment and Health Research Unit, South African Medical Research Council, Pretoria, South Africa; 2 Department of Geography, Geo-informatics and Meteorology, University of Pretoria, Pretoria, South Africa; 3 Cancer Society Social and Behavioural Research Unit, University of Otago, Dunedin School of Medicine, New Zealand; 4 Faculty of Health Sciences, University of Johannesburg, South Africa

**Keywords:** Sunbeds, indoor tanning, skin cancer, regulation, South Africa

## Abstract

**Introduction:**

In 2009, ultraviolet-emitting tanning devices, i.e. sunbeds and tanning booths, were officially classified as carcinogenic to human health (Group 1) by the International Agency for Research on Cancer.

**Methods:**

Here, we aim to estimate South African-based facilities with indoor tanning services advertised in the printed Yellow Pages and online in two directories. Printed Yellow Pages telephone directory beauty salon facilities listings (2010-14) for all provinces were examined and those mentioning "sunbed" and/or "tanning bed" recorded. Beauty/spa facilities were also identified using two sunbed listing webpages.

**Results:**

A total of 40 web-advertised facilities had a sunbed. Beauty facilities in the Yellow Pages specifically mentioning sunbeds declined by 62% between 2010 (n=53) and 2014 (n=20). Gauteng had the highest number of facilities (n=25) with a sunbed. Facilities with sunbeds exist in South Africa, as evidenced by the Yellow Pages and web-advertised data, but their true prevalence remains largely unknown. It is likely that online and walk-by advertising is increasingly more common than print.

**Conclusion:**

Given that sunbeds may likely soon become regulated in South Africa, further research is needed to better quantify sunbed provision, determine advice provided by facility operators to new users, investigate whether age restrictions or limits exist for sunbed use, and describe typical patterns of sunbed use in South Africa.

## Introduction

Sunbed use is a risk factor for skin cancer, including cutaneous malignant melanoma (CMM), basal cell carcinoma (BCC) and squamous cell carcinoma (SCC) [1]. In 2009, the International Agency for Research on Cancer (IARC) classified sunbeds as "carcinogenic to humans" [2]. The World Health Organization (WHO) recommends against the use of tanning devices for cosmetic purposes, but recognized the need for guidance to reduce the risks associated with their widespread use [3]. Several jurisdictions have implemented regulatory control of sunbed use, [4] including the city of Auckland in New Zealand [5] and all states in Australia, except the tropical Northern Territory, which has no sunbeds [6]. Controls implemented include a range of strategies from an outright ban, through ensuring the training of operators to warning signs and labels disclosing the associated risks [3]. Full bans have been implemented in Brazil and Iran [4]. Age limit bans exist in several jurisdictions of Canada as well as in Finland, Denmark, France, Germany, Spain and several other countries according to the worldwide sunbed legislation database [4].

In South Africa, skin cancer accounts for one-third of all histologically-diagnosed cancers [7]. Personal exposure to solar ultraviolet radiation (UVR) from the sun is a known risk factor for skin cancer; other risk factors include fair skin, a history of sunburns, dysplastic moles, precancerous skin lesions, a family history of skin cancer and a person history of skin cancer [8]. Africa experiences relatively high solar UVR levels almost all-year round. High UVR levels combined with an outdoor lifestyle may account, in part, for the relatively high incidence of skin cancers in South Africa. In the Western Cape, for example, the age-standardised incidence rate of new registered cases of CMM is estimated to be as high as 69 per 100 000 among Caucasians [9]. Similar provincial data are not available for other parts of South Africa. Nationally, the highest annual incidences of BCC, SCC and CMM occur among the White (Caucasian) population group. Caucasians with fair skin are considered to be more likely to seek a suntan than other South African population groups, namely Coloureds, Indians/Asians and Blacks, among which lower rates of skin cancer are reported. For males, the national 2000-2004 mean age-standardized annual incidence of reported CMM in Black, Asian, Coloured and White groups was 1.0, 0.7, 4.1 and 20.5 per 100 000 people, respectively [7]. While we do not know whether CMM incidence, in part, in South Africa is attributable to sunbed use, international evidence suggests that this is likely [1]. Since 2013, the Cancer Association of South Africa has been working with the National Department of Health to support the implementation of sunbed regulations in South Africa.

Given that sunbed use is associated with skin cancer and there was anecdotal evidence that commercial sunbeds existed in South Africa, this raised the research question, "what is the prevalence of sunbeds in South Africa" At present, South Africa has no legislation or code of practice governing indoor tanning and associated vertical or horizontal equipment, and the number of sunbeds advertised as available for use in the indoor tanning industry was unknown. This study aimed to assess the prevalence of indoor tanning facilities with sunbeds at health and beauty facilities for commercial use by the public. This was done in two parts, to document: 1) from 2010 to 2014, the number of health and beauty facilities by province/region that specifically mention "sunbed" and / or "vertical sunbeds" (referred to collectively as sunbeds) in their Yellow Pages advertisements; and 2) by province, the number of facilities with sunbeds advertised in 2015 in two web listings of indoor tanning facilities. In so doing, an estimate of the number of facilities offering sunbed services was determined, for the first time, and, by comparison of the two datasets, differences in advertising patterns of indoor tanning facilities were explored.

## Methods

### Yellow Pages data

**Figure 1:**
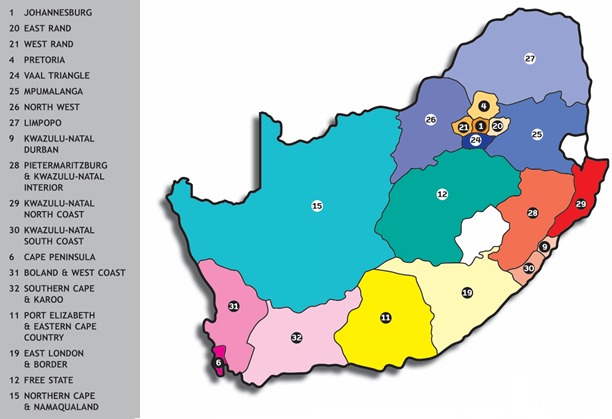
Map of South Africa illustrating the Yellow Pages regions. Lesotho and Swaziland are the two white countries on left and right, respectively. (Permission to use this graphic was obtained from its producer ‘Trudon Business’)

The printed (hard copy, not online Yellow Pages) beauty facilities pages from all 19 Yellow Pages-defined regions of South Africa (Figure 1) were provided with permission in pdf format from the manufacturer of the Yellow Pages in South Africa. There is no "Indoor Tanning Facilities" category in the South African Yellow Pages indexing. Data from 2010 to 2014 were provided as the Yellow Pages for these years were in digital format. A complete list of all Beauty Facilities was prepared in Microsoft Excel and included the facility name, physical address and telephone number. The province and / or region of the facility was also noted. Using MySQL, duplicates based on name, identical physical location and telephone number in the same year were removed from the dataset. This was followed by a complete list of those Beauty Facilities specifically mentioning in their Yellow Pages advertisement the word "sunbed" and/or "tanning bed". We were not able to receive the Yellow Pages data for Fitness centres and Hairdressers and this is noted as a shortcoming of the study for future research.

### Web-advertised data

Two online website listings specifically include an indexed category for indoor tanning facilities in South Africa. The website addresses are (1) http://www.saspaassociation.co.za and (2) http://thesalonguide.all4women.co.za. All of the facilities listed on both of these websites as at 1 October 2015 in the indoor tanning index were documented in a Microsoft Excel spreadsheet. The name, physical address and telephone number(s) of each facility was included in this list. Duplicates within and between the two website listings that had the identical physical location and telephone number were removed from the dataset. The facilities were also categorized by province.

### Analysis

The cleaned Yellow Pages data in MySQL database format and the web-advertised dataset from EpiData 3.1 (EpiData, Odense, Denmark) were merged, retaining their source as a variable, and imported into STATA I/C 13.1 (StataCop LP, USA) for further analysis. Summary descriptive statistics, including observed frequencies for all variables, were calculated.

## Results

### Yellow Pages

In 2010, a total of 53 facilities nation-wide specifically advertised as having sunbeds were listed in the Yellow Pages telephone directories (Table 1). Yellow Pages listings were available for all years and all regions, except for Pretoria in 2011 and 2013 (printed data were not available from the manufacturer of the Yellow Pages and we could not find a hard copy of the 2011 Yellow Pages book). The region with the highest number of facilities that advertised the availability of sunbeds was Region 11 in Figure 1, "Port Elizabeth and the Eastern Cape". For regions Mpumalanga (Region 25), North West Province (Region 26), Limpopo (Region 27), Vaal Triangle (Region 24), KwaZulu-Natal North Coast (Region 29) and KwaZulu-Natal South Coast (Region 30), there were no facilities that advertised the availability of sunbeds in the Yellow Pages listings. When analysed by year, between 2010 and 2014, the number of facilities that advertised they specifically had sunbeds as listed in the Yellow Pages decreased between 50% and 83% for seven and remained the same for 11 of the 19 regions. When the number of facilities which advertised in the Yellow Pages that they specifically had sunbeds was expressed as a percentage of the total number of facilities advertised in the Beauty Facility category, this percentage decreased 62%, nationally, from 53 in 2010 to 20 in 2014. Table 2 shows the regional distributions of sunbed facilities in South Africa with the numbers of "new" beauty facilities specifically mentioning sunbeds in their Yellow Pages listings for each year compared to the previous year/s. "New" facilities were considered to be facilities with a name different from previously listed facility names and at a different physical location than any of the facilities from the previous year. The years 2011, 2012 and 2013 saw the largest number of new businesses, but these numbers decreased over time, which is consistent with the observed overall decline in Yellow Pages listed facilities that specifically advertised sunbeds.

**Table 1:** Numbers of “beauty” facilities, and numbers and percentages of those specifically mentioning sunbeds, as advertised in the Yellow Pages in South Africa, by region and year, with percentage decrease, 2010-14 (where applicable).

	Total	With Sunbed	Total	With Sunbed	Total	With Sunbed	Total	With Sunbed	Total	With Sunbed	% decrease
	2010	2010	2011	2011	2012	2012	2013	2013	2014	2014	2010-14
**Region (Number onFigure 1)**											
Johannesburg (1)	164	4	171	3	167	3	158	1	159	1	75
Ekurhuleni (East Rand) (20)	128	6	129	3	129	3	129	3	110	1	83
West Rand (21)	0	0	64	3	63	1	69	1	57	0	-
Pretoria (4)	147	3	*	*	133	1	*	*	137	137	-
Vaal Triangle (24)	23	0	26	0	33	0	34	0	37	0	-
Mpumalanga (25)	48	0	43	0	40	0	42	0	41	0	-
North West (26)	32	0	34	0	34	0	35	0	31	0	-
Limpopo (27)	14	0	15	0	18	0	23	0	21	0	-
KwaZulu-Natal (Durban) (9)	264	1	270	2	284	2	92	1	301	1	0
Pietermaritzburg and KwaZulu-Natal Interior (28)	61	1	68	1	71	1	73	1	65	1	0
KwaZulu-Natal North Coast (29)	39	0	41	0	40	0	47	0	43	0	-
KwaZulu-Natal South Coast (30)	20	0	20	0	22	0	26	0	24	0	-
Cape Peninsula (6)	254	7	270	3	284	3	307	3	308	3	57
Boland and West Coast (31)	109	6	116	2	119	0	128	1	128	1	83
Southern Cape and Karoo (32)	60	2	56	1	63	1	74	1	66	2	0
Port Elizabeth and Eastern Cape (11)	99	14	86	11	90	10	111	10	124	7	50
East London (19)	40	4	41	2	31	3	45	4	47	1	75
Free State (12)	44	4	43	4	45	3	43	2	34	1	75
Northern Cape and Namaqualand (15)	31	1	33	0	37	1	39	1	38	1	0
**Total**	**1577**	**53**	**1526**	**35**	**1703**	**32**	**1475**	**30**	**1771**	**20**	**62**
Facilities with sunbeds compared to total number of facilities (%)		3.36		2.29		1.87		2.03		1.12	-

Note. * Missing data

**Table 2:** yellow pages regional data (2010-2014) for numbers of new “beauty” facilities specifically mentioning sunbeds for each year compared to the previous year/s.

	Businesses in 2010	Newbusinesses in year **2011** compared to previous year (2010)	New businesses in year **2012** compared to previous year (2011)	Newbusinesses in year **2012** compared to previous years (2011 – 2010)	Newbusinesses in year **2013** compared to previous year (2012)	Newbusinesses in year **2013** compared to previous years (2012 -2010)	Newbusinesses in year **2014** compared to previous year (2013)	New businesses in year **2014** compared to previous years (2013- 2010)
**Number of new beauty facilities with sunbed**	53 (total number in 2010)	9	6	2	7	6	2	2
**Regions**	(see Table 1)	1 x Port Elizabeth and Eastern Cape 1 x Cape Peninsula 3 x West Rand 1 x Johannesburg 1 x Boland and West Coast 1 x KwaZulu-Natal (Durban) 1 x Free State	2 x Port Elizabeth and Eastern Cape 1 x East London 1 x Ekurhuleni (East Rand) 1 x Northern Cape and Namaqualand 1 x Pretoria	1 x Port Elizabeth and Eastern Cape 1 x East London	2 x Port Elizabeth and Eastern Cape 1 x East London 1 x West Rand 1 x Ekurhuleni (East Rand) 1 x Boland and West Coast 1 x Free State	2 x Port Elizabeth and Eastern Cape 1 x East London 1 x West Rand 1 x Ekurhuleni (East Rand) 1 x Free State	1 x Southern Cape and Karoo 1 x KwaZulu-Natal (Durban)	1x Southern Cape and Karoo 1 x KwaZulu-Natal (Durban)

### Web-advertised

A total of 244 facilities were identified from the two web listings of indoor tanning businesses. Provinces with the largest numbers of web-advertised facilities were Gauteng (n=134), Western Cape (n=51) and KwaZulu-Natal (n=33). Overall, 40 of these facilities advertised that they had a sunbed, with Gauteng having the highest number (n=25). A cross-check was carried out to compare the names, physical locations and telephone numbers of the web-advertised facilities confirmed as having sunbeds with comparable information about of the beauty facilities specifically mentioning in their printed Yellow Pages advertisements that they had sunbeds. Results (Table 3, column 3) showed that only three of the web-advertised facilities also advertised in the printed Yellow Pages advertisements in any of the provinces in South Africa.

**Table 3:** Number of web-advertised “beauty” facilities with sunbeds

Province	Number of web-advertised facilities n	Number of web-advertised facilities with sunbeds n	Number of identical facilities advertised in the 2014 Yellow Pages and online n	Mid-year population estimates by province for 2014 % of total population [18]
Gauteng	134	25	0	23.9
Limpopo	6	5	0	10.4
Mpumalanga	5	0	0	7.8
Free State	5	1	0	5.2
Western Cape	51	3	1	11.3
KwaZulu-Natal	33	4	1	19.8
Eastern Cape	8	1	1	12.6
North West	2	1	0	6.8
Northern Cape	0	0	0	2.2
**Total**	244	40	3	100.0

Note: [18] see references

## Discussion

Unequivocal evidence supporting the association between sunbed use and skin cancer, together with the IARC classification of sunbeds as carcinogenic to human health, have led to an increase in sunbed surveillance [10, 11], regulation [12] and research [13, 14]. No study has reported sunbed distribution in South Africa, to date. The Yellow Pages audit followed Makin et al [15] and Jopson and Reeder [16]. The latter mentioned "brief internet Google searches identified examples of premises that offered indoor tanning services but which were not listed under the Yellow Pages categories as offering these services" (page 375) [16] therefore we included an objective to try to assess Internet-advertised beauty facilities with sunbeds in South Africa.

In response to objective 1), we found that the number of facilities offering sunbed services in South Africa advertised in the printed Yellow Pages was relatively low (the calculated rate per 100 000 Caucasian population was 1.2). International comparisons are problematic, but in 2009 there were an estimated 8 000 tanning salons (including sunbeds, spray tanning etc.) in the UK amounting to a availability of ~ 12.8 sunbed facilities per 100 000 total population [17]. New Zealand had 545 indoor tanning facilities (12.0 facilities per 100 000 total population, calculated using the number of indoor tanning facilities reported in the article and the number of New Zealand citizens in 2011 (n = 4 403 000) as reported by Statistics New Zealand) [18] listed in the Yellow Pages in 2006 [16]. One likely reason for the relatively low numbers of facilities which provide sunbeds in South Africa is population demographics. Of the total South African population (n = 54 002 000 in 2014), 80.2% are Black African, 8.8% are Coloured, 2.5% are Indian/Asian and 8.4% are White [19]. Sunbed users usually aim to tan their skin. White (Caucasian) individuals are most likely to desire a suntan compared to other population groups in South Africa. Given the relatively small White subpopulation in the country, supply and demand may lead to fewer sunbeds being available compared to countries with majority White populations. Caucasian individuals in South Africa may also use natural sunlight for sun tanning purposes given the predominantly sunny South African climate experienced across much of the country. Most parts of the country receive on average more than 2 500 hours of sunshine per year [20].

Since we were able to access 5 years of printed Yellow Pages data, we explored the change in sunbed advertising between 2010 and 2014. Electronic advertising and consumer use of the Internet for commercial purposes and services has increased significantly in recent years [21]. This appeared to be confirmed in the printed Yellow Pages data, which showed a decline in the number of facilities with sunbeds, 2010-2014. The cost of advertising may also have contributed to this observed reduction, although this is unconfirmed and perhaps an insignificant factor. It is unknown whether some of these facilities stopped operating for other reasons, such as changing consumer demands, negative publicity in other countries around sunbed use, and economic pressures. Previously published studies show large annual increases in the prevalence of sunbeds and similar indoor tanning industry services, but those studies were carried out in the 1990s and early 2000s. In the digital age, with the use of electronic searching for product and services, it is likely that the printed Yellow Pages are less utilised now by businesses compared to previous decades.

Regarding objective 2, there were twice as many facilities with sunbeds advertised online in 2015 compared to the total number of facilities advertised in the Yellow Pages in 2014. Furthermore, only three of the facilities advertised in the printed Yellow Pages in 2014 were listed online in the two databases of facilities supposedly with indoor tanning services accessed in September / October 2015. The province with the highest number of facilities with sunbeds was Gauteng. This province has the largest proportion of the population (23.9%) (Table 3, column 4) and also among the highest average household expendable income [22]. An increased proportion of business advertising budgets is likely to be allocated to online advertising. Gauteng is one of the landlocked provinces in the country; and it is well known that families and individuals from Gauteng regularly travel to coastal provinces to visit the beaches. There is a possibility that, since there is no beach in Gauteng, sunbeds are an alternative option to acquire a tan before a summer holiday at the coast, or when people cannot go to the beach or on vacation to acquire a tan.

This study protocols did not include telephoning all of the listings to confirm whether or not they did have a sunbed as specified in their advertisement. The study also only focused on sunbeds; none of the other indoor tanning services, such as spray tanning, were included since the study rationale focused on the risk of sunbed use in relation to skin cancer development. The study did not consider commercial sales or hire of sunbeds, however, these services do exist in South Africa, for both commercial and home-based use. We were not able to access from the company the printed Yellow Pages data for "health and fitness centres" or hairdressers where sunbeds may be available because some of these data were not digital and required resources which we did not have in order to be scanned. We did note several facilities advertised in the online listings were located inside a gym or fitness centre.

This study is likely to have underestimated sunbed prevalence in South Africa. It is possible that advertising of indoor tanning facilities is often opportunistic with store front signage to attract drive-by or walking customers. The precise number of sunbeds available for commercial use in South Africa remains unknown and is likely to remain so unless mandatory registration of such premises and operators is introduced. It is unlikely that tertiary institutions offering health, beauty and somatology courses provide any training on sunbed operation; one college confirmed that they did not provide training on sunbed operation as part of their coursework and practical teaching. Usual operator guidance and advice regarding sunbed use in South Africa as well as the patterns of sunbed use and characteristics of sunbed users (known for other parts of the world) [23] remain unknown.

## Conclusion

In South Africa, advertisements in the printed Yellow Pages have declined, but been overtaken by Internet advertising, which is more difficult to monitor, therefore we still know little about the prevalence and use of indoor tanning facilities in South Africa. Little is known about the manner of sunbed use, existing guidelines, client profiles and advice given to those making use of sun tanning facilities. All of these are important for decision and policy making. Further research is needed to better estimate sunbed prevalence, analyse the advice provided by facility operators to new users, determine whether age restrictions or limits exist for sunbed use and describe the typical patterns of sunbed use (and user characteristics) in South Africa in order to be able to better assess sunbed-related health risks and associations with skin cancer outcomes. The World Health Organization recommends that facilities offering sunbed services should provide appropriate information to consumers, restrict access only to those 18 years or older. They also recommend ensuring that tanning establishments should have operator surveillance and that unsupervised, self-service sunbeds be banned [3]. There is currently little or no regulation and /or monitoring of sun tanning facilities in South Africa, despite the known risks. This may change should the proposed sunbed regulations be promulgated. The implementation of sunbed regulations in South Africa may help reduce the relatively high incidence of skin cancer observed among Caucasian individuals, however, personal excess exposure to the sun is likely to remain an important risk factor and appropriate sun safety practices should be followed.

### What is known about this topic

- Sunbed use is a risk factor for skin cancer;
- Several countries and jurisdictions have implemented regulatory control of sunbed use or outright sunbed bans.

### What this study adds

- A baseline of sunbed prevalence has been provided ahead of possible implementation of sunbed regulations in South Africa;
- Despite interrogating sunbed advertising as best we could, we still know little about the prevalence and use of indoor tanning facilities in South Africa;
- Further sunbed use and sun exposure research is recommended to combat skin cancer in South Africa.
